# Advances in sports nutrition, exercise and medicine: Olympic issues, the legacy and beyond

**DOI:** 10.1186/1741-7015-10-79

**Published:** 2012-07-19

**Authors:** Mike Carmont

**Affiliations:** 1 The Princess Royal Hospital, Shrewsbury & Telford NHS Trust and the Northern General Hospital, Sheffield Teaching Hospitals NHS Foundation Trust, Sheffield, UK

## Abstract

In the run up to the London 2012 Olympics, this editorial introduces the cross-journal article collection Advances in Sports Nutrition, Exercise and Medicine http://www.biomedcentral.com/series/asnem

## Editorial

Since 2006, Sports and Exercise Medicine (SEM) in the United Kingdom has developed from General Practitioners with specialist interests and those from other allied specialties, to a specialization within its own right and the formation of its own Faculty. The progression of this specialty has gone hand in hand with preparations for the Olympic Games in London commencing 27^th ^July 2012.

Now, in the run up to the Games, SEM practitioners are completing preparations for the competition itself and are starting to look towards the continued care of the athletes and teams.

Within the UK, recent cardiac events have resulted in both tragedy and successful resuscitation. The cardiac arrest of a Premier League Footballer during a match has led to calls for the cardiac screening of all professional footballers in the UK. Italian studies have shown that the screening of athletes for the presence of cardiac abnormalities prior to sports participation has led to a reduction in mortality in this cohort [1-3].

For players with no family history or detectable underlying cardiac abnormality, screening may not have prevented this cardiac arrest. The quick response of the team doctors and physiotherapists trained, practiced and prepared for cardiac events at the pitch side without doubt lead to improved outcome. It is well known that there is a 10% reduction in outcome for each minute that passes before defibrillation [4]. Good quality cardiopulmonary resuscitation is a skill, which everyone can learn and this preserves perfusion to the brain [5]. There has also been discussion as to whether resuscitation should be performed on the pitch. While, these scenes may appear distressing for the spectators, not witnessing this care may be more distressing in the long term.

The tragic death of a school-girl hit in the chest by a rugby ball has highlighted discrepancies between the provision of emergency care on the school playing field and the professional game. This rare event, *Commotio cordis*, occurs when the force of the impact occurs precisely on the upstroke of the T wave when the heart is repolarising and an arrhythmia develops [5]. It was recently appreciated that it is not the force of this impact but the unfortunate precision of the impact [6]. Once again screening would not have prevented this as *Commotio cordis *occurs in hearts that are structurally and electrophysiologically normal. Prompt defibrillation, if available, even by bystanders, can restore cardiac output [7]. It may be, however, that the adoption of a screening programme together with the availability of defibrillators at sporting events is the optimal course of prevention and preparation for sudden cardiac events during sports [3,8].

Preparation also includes the avoidance of injury, which was the subject of a systematic review [9] reviewing a number of neuromuscular warm up strategies to prevent lower limb and knee injuries. The 11+, Prevent injury and Enhance Performance (PEP), Harmoknee and Knee Injury Prevention Programme (KIPP) all feature components of stretching, strengthening, balance, sports specific agility drills and landing techniques for periods of 3 months. It is hoped that injuries such as anterior cruciate ligament rupture and their sequelae will be minimized by the adoption of these programmes.

Over the course of the 2012 Olympic Games, we will witness athletes performing faster, higher and stronger than their predecessors. This may pose a couple of questions. Firstly, how far is an athlete prepared to go to get an advantage? Secondly, how much are they prepared to use and possibly abuse their bodies to win?

The impact of these effects can be observed both in the short and long term. The World Anti-Doping Agency is an international independent organization responsible for promoting, co-ordinating and monitoring the fight against doping in sport. The agency produces a list of banned substances each year called The Prohibited List [10]. It must be remembered that in every case the athlete has full responsibility for everything that goes into their body. Information about substances is readily available for athletes on sites such as 100 percentme [11] and the prohibited list is even available as an iphone application [12].

One legal way in which performance can be enhanced is by some forms of dietary supplementation. For instance, betaine supplementation has been shown to increase cycling sprint power in recreationally active males and females [13]. Moreover, the optimization of diet prior to commencing an exercise programme is important. Military recruits sustaining stress fractures during elite military training were found to have previous dietary deficiencies compared to those who were injury free [14]. However, those skeptical about the ability of sports drinks to rehydrate and improve performance will be interested in Kalman's recent study. After being dehydrated to a 2% reduction in body mass during a 60 minute bout of treadmill exercise, subjects showed little difference in a subsequent exercise to exhaustion test after rehydrating with bottled water, coconut water, coconut water concentrate or sports drink [15].

Many interventions can aim to enhance performance, but in the end, the athlete's efforts will be the culmination of hours of training and preparation. It is now appreciated that elite athletes may suffer long-term degenerative joint disease as a result of their hours of training [16]. The early management of these patients may well be able to postpone definitive joint replacement surgery. Symptom alleviation by intra-articular injections of corticosteroid, hyaluronic acid (HA) and platelet rich plasma (PRP) are attractive options.

Injections of HA may act as both a mechanical lubricant and act biologically to stimulate the synoviocytes to produce synovial fluid with improved the physical properties. Meta-analyses have suggested peak effectiveness at 8 weeks following injection and that there is still a residual detectable effect after 24 weeks [17]. HA injections are more effective than corticosteroid injections beyond 8 weeks following injection [18].

PRP injections are specific fractions of centrifuged autologous blood and may be injected to prevent deterioration and promote regeneration of the articular surface [19]. PRP releasate has been shown to inhibit the inflammatory processes in osteoarthritic chondrocytes and decreases activation in the pathways involved in the pathogenesis of osteoarthritis [20]. PRP injections have also been shown to provide a greater and longer efficacy than HA injections in reducing symptoms and the recovery of articular function [21]. It must be remembered that when discussing the effectiveness of PRP that there are numerous methods of preparation of PRP and preparations rich in growth factors. Thus treatment using PRP will vary considerably in different studies [22].

Strategies to promote recovery are not limited just to athletes. With joints frequently wearing out at a younger age when patients have busy, working, and productive lives, definitive strategies may have to be adopted. Degeneration may only occur in part of a joint e.g. the medial compartment of the knee. Re-alignment osteotomy may prolong the life expectancy of the patient's native joint by offloading this area [23] and may be comparable to a partial joint replacement [24]. Patients always ask about future function before surgery. Although patients may return to sporting activity, they do not perform at their previous level and residual pain is not exceptional [25]. Alternatively biological knee reconstruction may be considered as a viable option [26].

In a similar vein, the influence of the Games stretches to the general public, and not just the athletes involved. Its influence in promoting health and exercise is something that has been considered since the Games were awarded to the city of London in 2007 [27]. The "Legacy" of the Olympic Games is promoted to encourage sports participation in the home nation. In this way, it is hoped that the general population can receive the benefits of sports participation for health and exercise, and this legacy has been considered to involve both the sports' community and the general community [28]. Three Olympic Legacy centres for Sports and Exercise Medicine have been selected to receive funding from the government to build SEM services in London, Loughborough and Sheffield [29]. Considering the difficulty of the current economic situation, the allocation of resources to leisure activities could be considered to be an expensive luxury. However, the promotion of health and exercise may save money in the long term as there is likely to be a higher degree of physical activity, and therefore a lower incidence of morbidities associated with the sedentary lifestyle to which many of us have become accustomed. It should be noted, however, that the success of this legacy requires the public to take up the exercise challenge so that the benefits can be appreciated for both the individual and the nation [30].

## Author's information

MC is a Consultant Trauma and Orthopaedic Surgeon with an interest in lower limb sports surgery and sports medicine. He is volunteering to provide medical services for the Olympic Games. He is also the Guest Editor for the Advances in Sports Nutrition, Exercise and Medicine cross-journal article collection.

## Pre-publication history

The pre-publication history for this paper can be accessed here:

http://www.biomedcentral.com/1741-7015/10/79/prepub
